# Supplementary material for: HDAC3 inhibition as a therapeutic strategy in T-cell acute lymphoblastic leukemia via the TYK2-STAT1-BCL2 signaling pathway
Source: Front Immunol. 2026 Feb 4;17:1752727. doi: 10.3389/fimmu.2026.1752727 (PMC12913501; doi:10.3389/fimmu.2026.1752727)
Supplement: Supplementary file 1 [file DataSheet1.docx]

| **Supplemental Table 1. Prior chemotherapies of 28 patients with refractory/relapsed T-ALL.** | |
| --- | --- |
| Patient No. | Prior chemotherapies before a salvage reinduction regimen |
| 1 | HyperCVAD-A, HyperCVAD-B, HyperCVAD-A+MTX, |
| 2 | MOAP, HyperCVAD-A |
| 3 | CHOP, HyperCVAD-A, HyperCVAD-B |
| 4 | CDOLP, HyperCVAD-A, HyperCVAD-B, HyperCVAD-A, HyperCVAD-B |
| 5 | HyperCVAD-A🞨2 |
| 6 | HyperCVAD-A, HyperCVAD-B, HyperCVAD-A🞨2, VDCLP |
| 7 | VDLP, CAT, HD-MTX🞨2, VDLP, CAT, MT, Radiotherapy |
| 8 | HyperCVAD-A |
| 9 | CHOP |
| 10 | HyperCVAD-A |
| 11 | HyperCVAD-A, HyperCVAD-B, HyperCVAD-A, HyperCVAD-B, HyperCVAD-A,  HyperCVAD-B |
| 12 | HyperCVAD-A, HyperCVAD-B, HyperCVAD-A, VDLP, HD-MTX🞨2, HD-MTX-Ara-c, Radiotherapy |
| 13 | VDCLP |
| 14 | VDLP, HyperCVAD-A, HyperCVAD-B, HyperCVAD-A, HyperCVAD-B |
| 15 | VDCLP, HyperCVAD-A, HyperCVAD-B |
| 16 | VDCLP, HyperCVAD-A+MTX, HyperCVAD-B, HyperCVAD-A, HyperCVAD-B |
| 17 | VDCLP, HyperCVAD-A, HyperCVAD-B, HyperCVAD-A, Haplo-HCT |
| 18 | HyperCVAD-A, HyperCVAD-B, HyperCVAD-A, MTX, CAM, MSD-HCT |
| 19 | DOLP🞨2, HyperCVAD-A, HyperCVAD-B, HyperCVAD-A, COEP, Auto-HCT, VP🞨10, Hyper-CVAD-A, HyperCVAD-B, HyperCVAD-A, HyperCVAD-B |
| 20 | MTX+VP |
| 21 | COP, Hyper-CVAD-A, MOAEP, HyperCVAD-B, HyperCVAD-A, CLA-Ida, VDCLP |
| 22 | VICLP, VDCLP, HyperCVAD-A |
| 23 | VILP🞨2, Hyper-CVAD-A, HyperCVAD-B, HyperCVAD-A, MSD-HCT |
| 24 | VICP, CAG, MTX+L+Venatoclax |
| 25 | Hyper-CVAD-A, HyperCVAD-B, HyperCVAD-A, HyperCVAD-B, HyperCVAD-A, HyperCVAD-B, Haplo-HCT |
| 26 | VDCP |
| 27 | VDCLP |
| 28 | VDCLP, HyperCVAD-A |
| T-ALL, T-cell acute lymphoblastic leukemia; C, cyclophosphamide; D, daunorubicin; E, etoposide; H, homoharringtonine; I, idarubicin; L, L-asparaginase; M, mitoxantrone; MTX, methotrexate; P, prednisone; V, vincristine; O, vincristine; HD, high dose; A, cytarabine; Ara-c, cytarabine; CAG, aclarubicin, low-dose cytarabine, and granulocyte colony-stimulating factor; haplo-HCT, haploidentical donor hematopoietic cell transplantation; MSD-HCT, matched sibling donor hematopoietic cell transplantation. | |

**Supplemental Table 2. Primer sequences (5’–3’) for genes in this study.**

| Gene | Forward primer sequences | Reverse primer sequences |
| --- | --- | --- |
| *HDAC1* | CGCCCTCACAAAGCCAATG | CTGCTTGCTGTACTCCGACA |
| *HDAC2* | ATGGCGTACAGTCAAGGAGG | TGCGGATTCTATGAGGCTTCA |
| *HDAC3* | TCTGGCTTCTGCTATGTCAACG | CCCGGTCAGTGAGGTAGAAAG |
| *HDAC10* | AGTGCCCTAGAGTCCATCCAG | CACAGCGGTCACATCTTGCT |
| *JAK1* | CCACTACCGGATGAGGTTCTA | GGGTCTCGAATAGGAGCCAG |
| *JAK2* | TCTGGGGAGTATGTTGCAGAA | AGACATGGTTGGGTGGATACC |
| *JAK3* | CCTGATCGTGGTCCAGAGAG | GCAGGGATCTTGTGAAATGTCAT |
| *TYK2* | GAACCGGCTGTGTACCGTT | ACGTCATTCACAAACTCATGCTT |
| *STAT1* | CAGCTTGACTCAAAATTCCTGGA | TGAAGATTACGCTTGCTTTTCCT |
| *STAT2* | GAGCCAGCAACATGAGATTGA | GCCTGGATCTTATATCGGAAGCA |
| *STAT3* | CAGCAGCTTGACACACGGTA | AAACACCAAAGTGGCATGTGA |
| *STAT4* | TGTTGGCCCAATGGATTGAAA | GGAAACACGACCTAACTGTTCAT |
| *STAT5A* | GCAGAGTCCGTGACAGAGG | CCACAGGTAGGGACAGAGTCT |
| *STAT5B* | CAGAACACGTATGACCGCTG | CTGGAGAGCTACCATTGTTGG |
| *STAT6* | CGAGTAGGGGAGATCCACCTT | GCAGGAGTTTCTATCAAGCTGTG |
| *CDKN1A* | TGTCCGTCAGAACCCATGC | AAAGTCGAAGTTCCATCGCTC |
| *BCL2* | GGTGGGGTCATGTGTGTGG | CGGTTCAGGTACTCAGTCATCC |
| *BCL2L1* | GAGCTGGTGGTTGACTTTCTC | TCCATCTCCGATTCAGTCCCT |
| *MYC* | GTCAAGAGGCGAACACACAAC | TTGGACGGACAGGATGTATGC |
| *MCL1* | TGCTTCGGAAACTGGACATCA | TAGCCACAAAGGCACCAAAAG |
| *PIM1* | GAGAAGGACCGGATTTCCGAC | CAGTCCAGGAGCCTAATGACG |
| *PTPN2* | GAAGAGTTGGATACTCAGCGTC | TGCAGTTTAACACGACTGTGAT |
| *PTPN6* | TGAACTGCTCCGATCCCACTA | CACGCACAAGAAACGTCCAG |
| *CCND1* | GCTGCGAAGTGGAAACCATC | CCTCCTTCTGCACACATTTGAA |
| *CCND2* | ACCTTCCGCAGTGCTCCTA | CCCAGCCAAGAAACGGTCC |
| *CCND3* | TACCCGCCATCCATGATCG | AGGCAGTCCACTTCAGTGC |
| *GAPDH* | GGAGCGAGATCCCTCCAAAAT | GGCTGTTGTCATACTTCTCATGG |

| **Supplemental Table 3. Salvage reinduction regimens and clinical responses of 28 patients with refractory/relapsed T-ALL.** | | |
| --- | --- | --- |
| Patient No. | Reinduction regimen | Response |
| 1 | Chidamide + MOEP-L | PR |
| 2 | Chidamide + Hyper-CVAD-B | CR |
| 3 | Chidamide + VDCLP | PR |
| 4 | Chidamide + VDCLP | PR |
| 5 | Chidamide + MOEP | CR |
| 6 | Chidamide + MOAP | NR |
| 7 | Chidamide + VDCLP | CR |
| 8 | Chidamide + VICLP | CR |
| 9 | Chidamide + VHICLP | NE |
| 10 | Chidamide + VDCLP | CR |
| 11 | Chidamide + VDCLP | CR |
| 12 | Chidamide + VDCLP | CR |
| 13 | Chidamide + VDCLP | CR |
| 14 | Chidamide + VHDCLP | NR |
| 15 | Chidamide + VDCLP | CR |
| 16 | Chidamide + VDCLP | NE |
| 17 | Chidamide + EPOCH | NR |
| 18 | Chidamide + 6-MP | NR |
| 19 | Chidamide + Hyper-CVAD-B | CR |
| 20 | Chidamide + COP | NR |
| 21 | Chidamide + VDCLP | NR |
| 22 | Chidamide + Hyper-CVAD-B | CR |
| 23 | Chidamide + Hyper-CVAD-A | NR |
| 24 | Chidamide + IOCLP + Venatoclax | CR |
| 25 | Chidamide + MTX + Ara-C + Dexamethasone | CR |
| 26 | Chidamide + VDCLP | CR |
| 27 | Chidamide + Hyper-CVAD-A + Decitabine | CR |
| 28 | Chidamide + Hyper-CVAD-B | CR |
| C: cyclophosphamide; O, vincristine; D: daunorubicin; E: etoposide; H: homoharringtonine; I: idarubicin; L: L-asparaginase; M: mitoxantrone; MTX: methotrexate; P: prednisone; V: vincristine; HD: high dose; Ara-c, cytarabine; CR: complete remission; PR: partial remission; NR: non-remission; NE: not evaluated. | | |

| **Supplemental Table 4. Frequency of adverse effects during salvage reinduction therapy.** | | |
| --- | --- | --- |
| **Adverse events** | **Cases** | **Percentage (%)** |
| Hypocellular bone marrow | 28 | 100 |
| Anemia | 28 | 100 |
| Febrile neutropenia | 14 | 50 |
| Liver injury | 5 | 17.9 |
| Oral mucositis | 5 | 17.9 |
| Diarrhea | 3 | 10.7 |
| Hypofibrinogen | 3 | 10.7 |
| Pneumonitis | 3 | 10.7 |
| Rash | 2 | 7.1 |
| Fatigue | 4 | 14.3 |
| Sepsis | 3 | 10.7 |
| Acute renal injury | 1 | 3.6 |

**Figure S1**


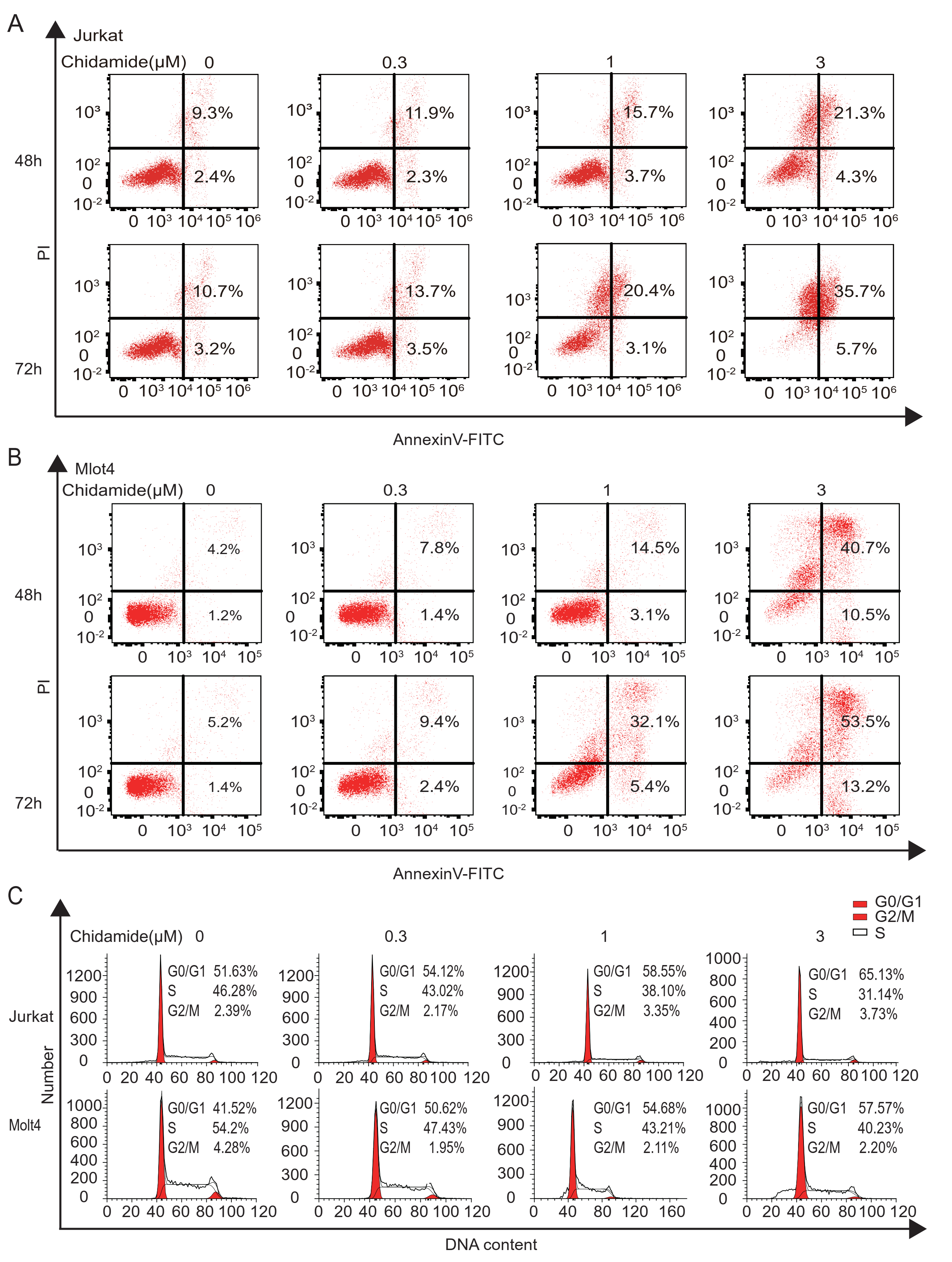


**Figure S1.** Chidamide inhibited the proliferation of T-ALL cells and induced apoptosis and cycle arrest in vitro. Chidamide induced apoptosis and cycle arrest of T-ALL cells in vitro. Jurkat and MOLT-4 cells were cultured with different concentrations of chidamide for 24, 48, and 72 h. **A** Representative results of apoptosis analyzed by flow cytometry in Jurkat cells. **B** Representative results of apoptosis analyzed by flow cytometry in MOLT-4 cells. **C** Representative results of G0/G1 phase and S phase proportions in response to incubation with chidamide for 24 h, measured with flow cytometry. The data represent three independent experiments. Results are shown as mean ± SD (*P < 0.05, **P < 0.01, ***P < 0.001, NS: P > 0.05).

**Figure S2**


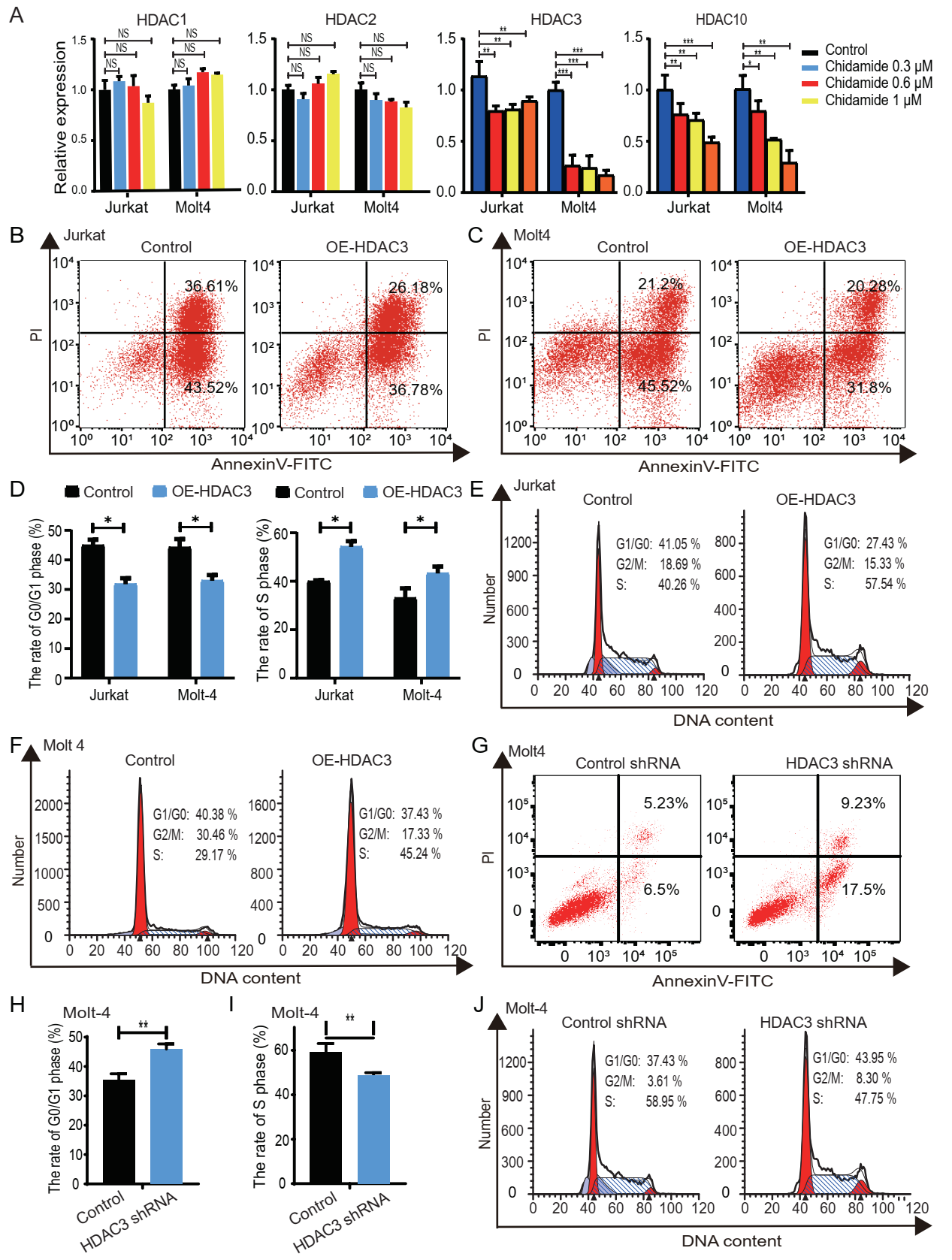


**Figure S2.** The inhibitory effect of chidamide on T-ALL cells was dependent on HDAC3. **A** The expression of *HDAC1*, *HDAC2*, *HDAC3*, and *HDAC10* in Jurkat and MOLT-4 cells after treatment with chidamide was measured by RT-PCR. Both Jurkat and MOLT-4 cells were transfected with overexpressing *HDAC3* plasmids or control plasmids (annotated as OE-HDAC3). The transfected cells were treated with 1 μM chidamide for 24 h. Overexpression of HDAC3 in response to chidamide is shown in **B–F**. Evaluation of apoptosis by flow cytometry. Representative results of apoptosis analyses are shown in Jurkat **(B)** and MOLT-4 cells **(C)**. The proportion of G0/G1 phase and S phase in Jurkat and MOLT-4 cells in response to incubation with chidamide for 24 h was measured with flow cytometry **(D)**. Representative results of G0/G1 phase and S phase proportions are shown in **E** and **F**. Transfection with *HDAC3* shRNA was performed in Molt4 cells **(G–J)**. Then, apoptosis was measured by flow cytometric analysis. Representative results of the apoptosis analysis are shown for MOLT-4 cells **(G)**. The proportion of G0/G1 phase and S phase was measured with flow cytometry **(H** and **I)**. **J** Representative results of the proportion of cells in the G0/G1 phase and S phase after *HDAC3* knockdown. The data represent three independent experiments. Results are shown as mean ± SD (*P < 0.05, **P < 0.01, ***P < 0.001, NS: P > 0.05).

**Figure S3**
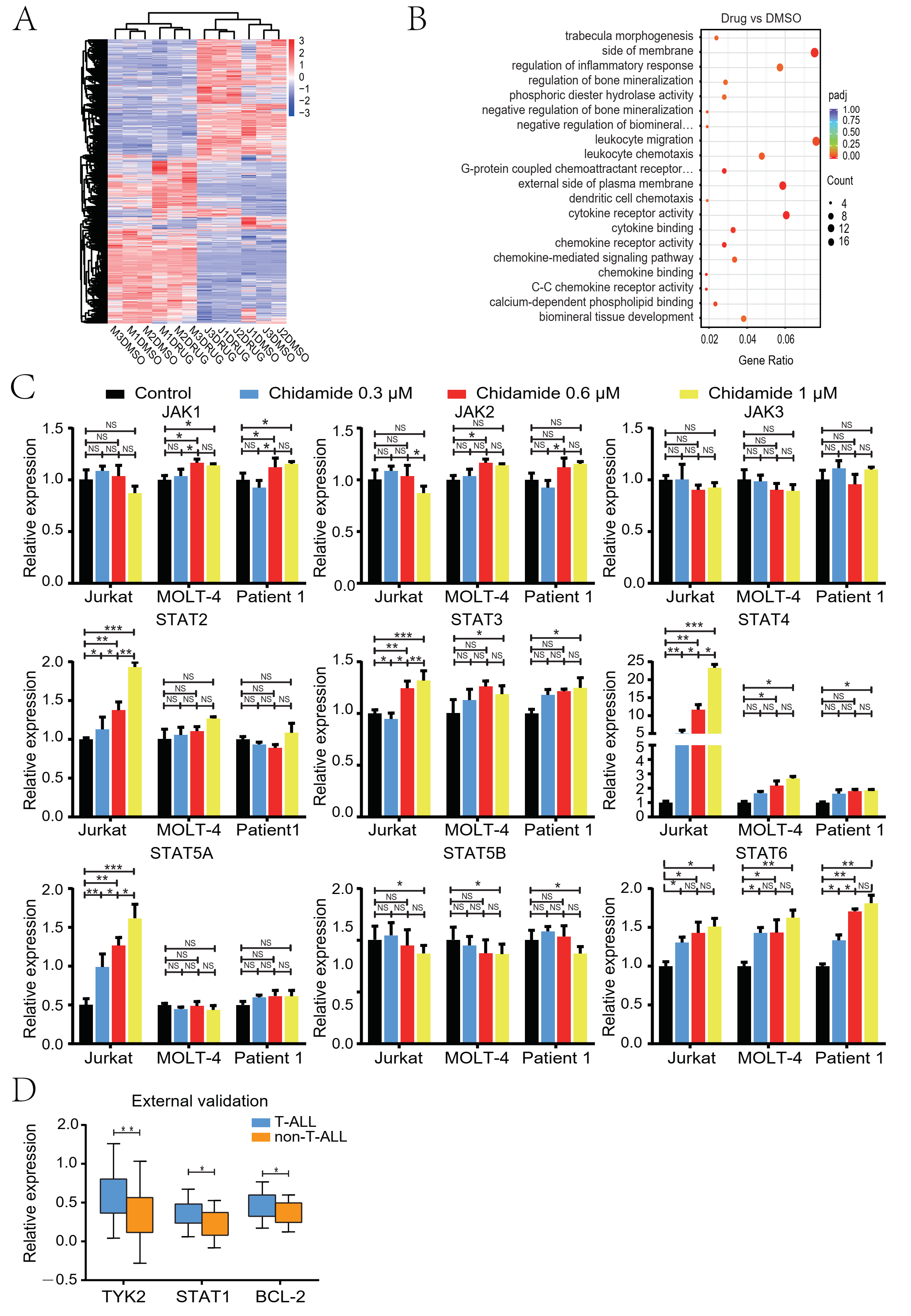


**Figure S3.** Differential gene and protein expression in T-ALL cells upon chidamide treatment and external validation using a public dataset. Total RNA isolated from T-ALL cell lines, which were treated with DMSO and chidamide, was subjected to RNA-sequencing. **A** Cluster analysis of differentially expressed genes upon chidamide treatment. **B** Gene Ontology (GO) biological process analysis of differentially expressed genes. **C** Differential gene expression was measured by RT-PCR in both T-ALL cell lines and primary samples. The data represent three independent experiments. **D** External validation of TYK2-STAT1-BCL2 axis–related gene expression using an independent public dataset from the NCBI Gene Expression Omnibus (GEO; GSE7186). Expression levels of TYK2, STAT1, and BCL2 were analyzed across leukemia subtypes. Results are shown as mean ± SD (*P < 0.05, **P < 0.01, ***P < 0.001, NS: P > 0.05).

**Figure S4**


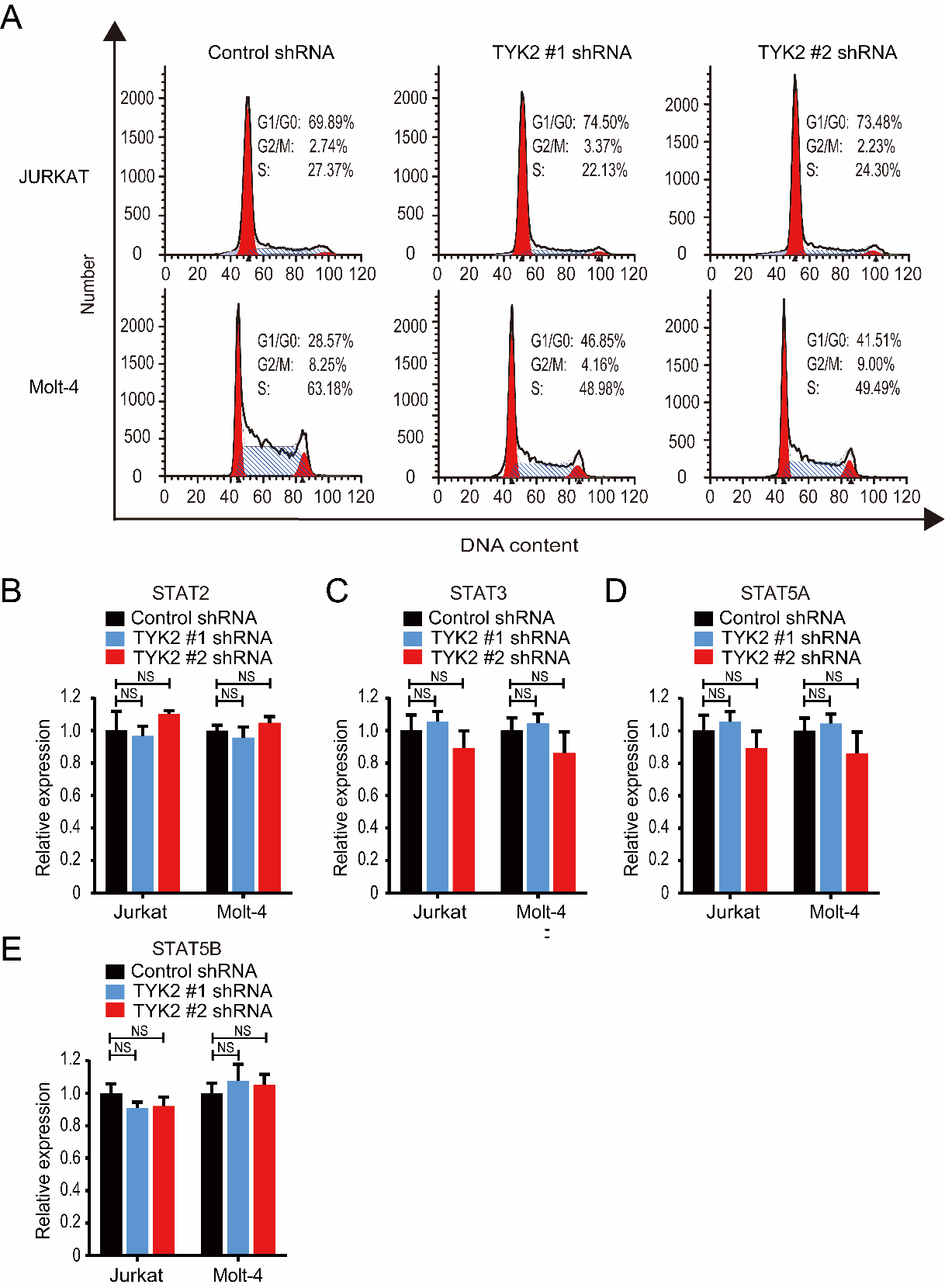


**Figure S4.** *TYK2* silencing reduced the induced cell cycle arrest of T-ALL cells. Transfection with *TYK2* shRNA was performed in Jurkat and Molt4 cells. The proportion of cells in the G0/G1 phase and S phase after *TYK2* knockdown was measured with flow cytometry. **A** Representative results of the proportion of G0/G1 phase and S phase cells after transfection. **B–E** The expression of *STAT2*, *STAT3*, *STAT5A*, and *STAT5B* after transfection with *TYK2* shRNA was measured by RT-PCR. The data represent three independent experiments, and the results are shown as mean ± SD (NS: P > 0.05).

**Figure S5**


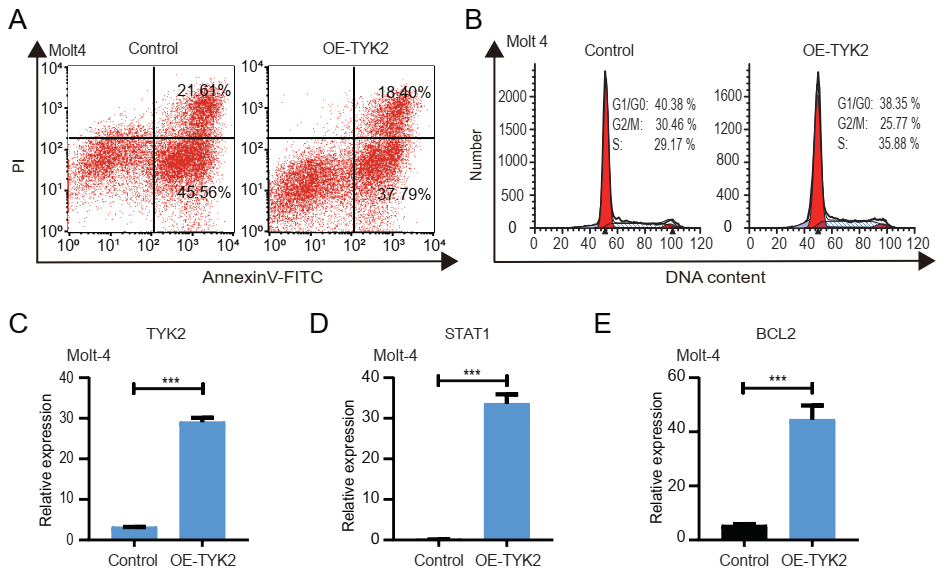


**Figure S5.** Overexpression of *TKY2* rescued the inhibitory effect of chidamide via the TYK2-STAT1-BCL2 signaling pathway. Both cell lines were transfected with overexpressing *TYK2* (annotated as OE-TYK2) plasmids or control plasmids. Transfected cells were treated with 1 μM chidamide for 24 h. **A** Representative results of cell apoptosis by flow cytometry in MOLT-4 cells. **B** Representative results of the proportion of G0/G1 phase and S phase cells after TYK2 overexpression. **C–E** The expression of *TYK2*, *STAT1*, and *BCL2* after transfection was measured by RT-PCR in MOLT-4 cells. The data represent three independent experiments, and the results are shown as mean ± SD (***P < 0.001).

**Figure S6**


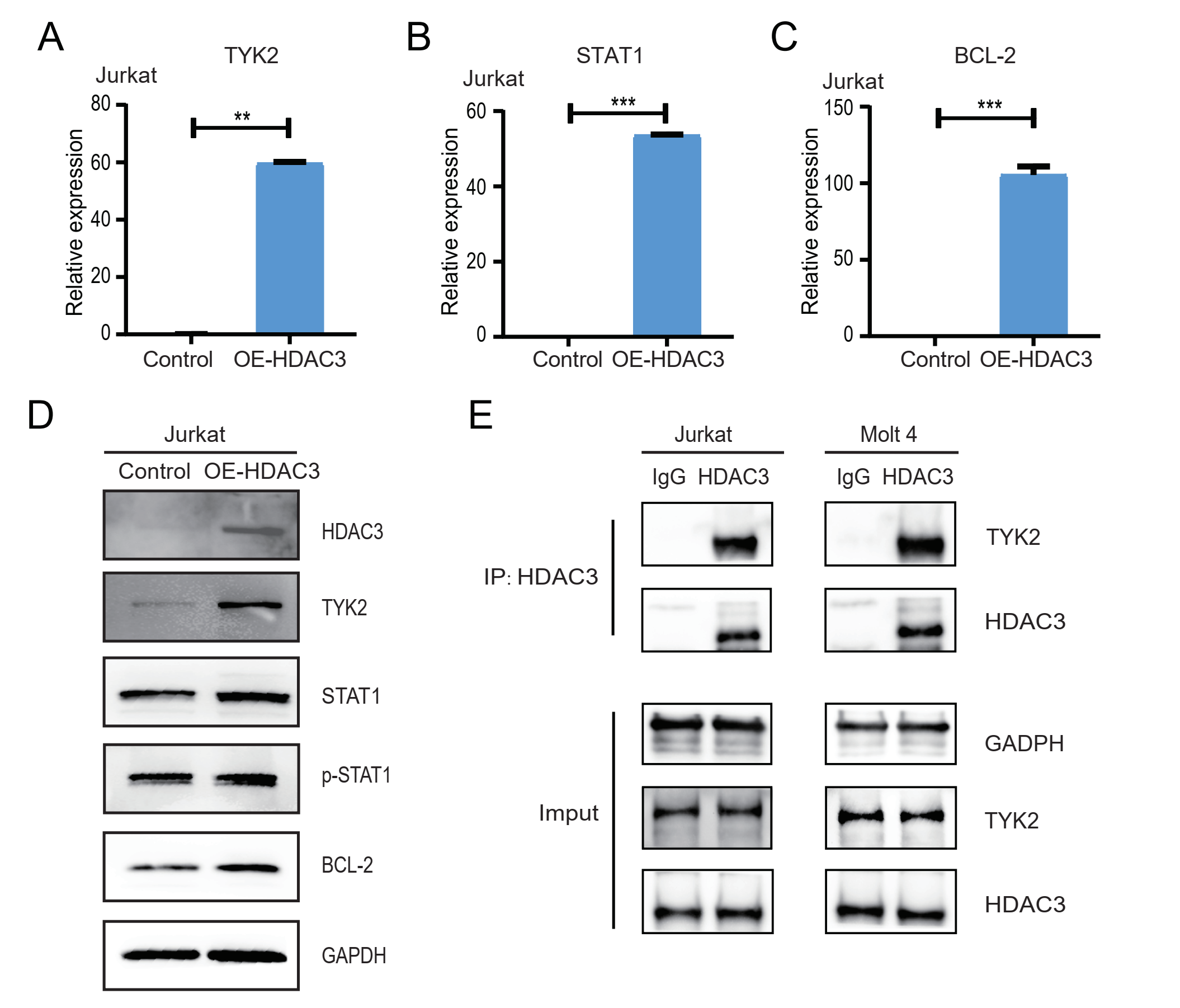


**Figure S6.** The inhibitory effect of chidamide on T-ALL cells was dependent on the HDAC3-TYK2-STAT1-BCL2 signaling pathway. **A–D** Jurkat cells were transfected with overexpressing *HDAC3* (annotated as OE-HDAC3) plasmids or control plasmids. The mRNA expression level of *TYK2* **(A)**, *STAT1* **(B)**, and *BCL2* **(C)** after transfection was detected by RT-PCR. **D** The expression of TYK2, STAT1, p-STAT1, and BCL2 after transfection was evaluated by Western blot. **E** Co-immunoprecipitation of TK2 and HDAC3 was performed using Jurkat and Molt-4 cells. The data represent three independent experiments. Results are shown as mean ± SD (*P < 0.05, ***P < 0.001, NS: P > 0.05).

**Figure S7**


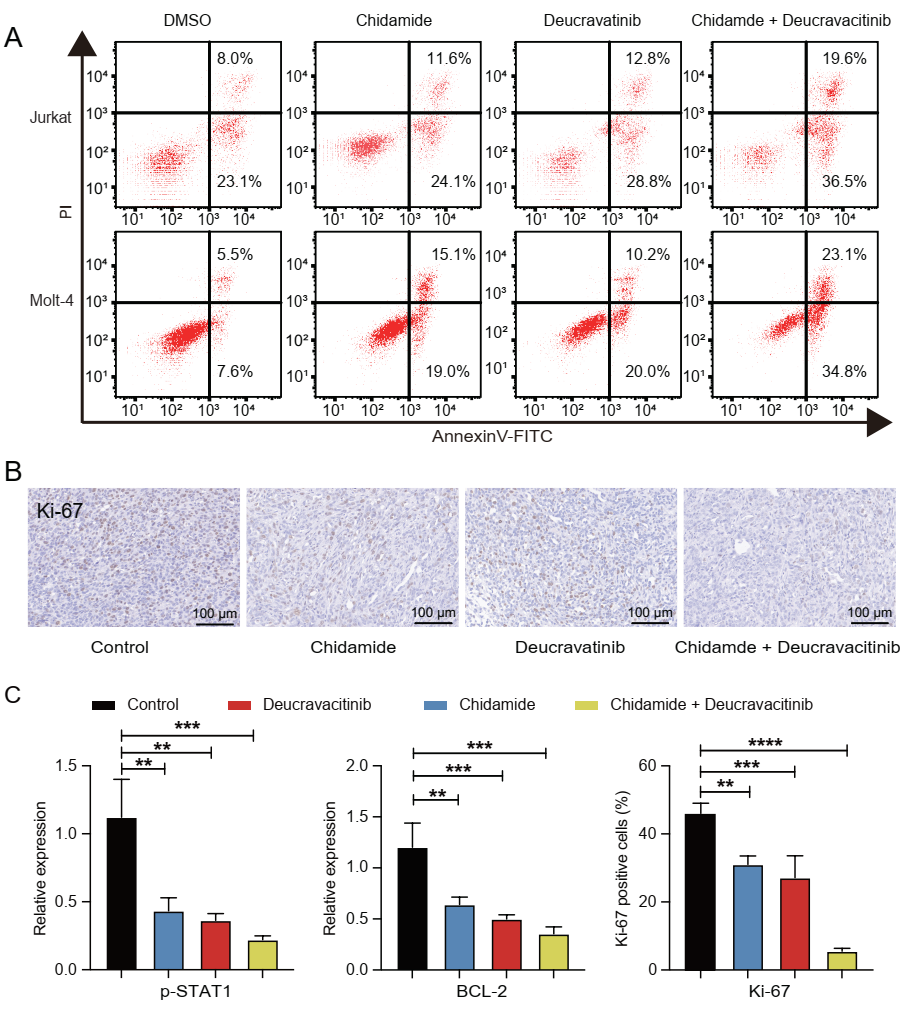


**Figure S7.** Chidamide and a Tyk2 inhibitor synergistically inhibited the growth of T-ALL cells both in vitro and in vivo. Jurkat and Molt-4 cells were incubated with 1 μM chidamide or 4 μM deucravacitinib as a monotherapy or with chidamide and deucravacitinib combined for 36 h. **A** Flow cytometry was used to analyze cell apoptosis. Representative results of flow cytometry plots for detecting apoptosis. Jurkat cells (1 × 10^7^ cells) were implanted into NOD/SCID mice. The mice were randomly divided into four groups (six mice in each group). The mice in the chidamide-treated group were intragastrically administered with chidamide three times a week for 2 weeks. The mice in the deucravacitinib-treated group were intragastrically administered deucravacitinib twice daily for 2 weeks. The mice in the control group were treated with both PBS and normal saline as a control. **B** Representative expression of Ki-67 in tumor sections by immunohistochemistry. **C** Relative expression levels of p-STAT1, BCL2, and Ki-67in tumor sections detected by immunohistochemical analysis. The data represent three independent experiments. Data are expressed as mean values ± SD (*P < 0.05, **P < 0.01, ***P < 0.001, NS: P > 0.05).

**Figure S8**


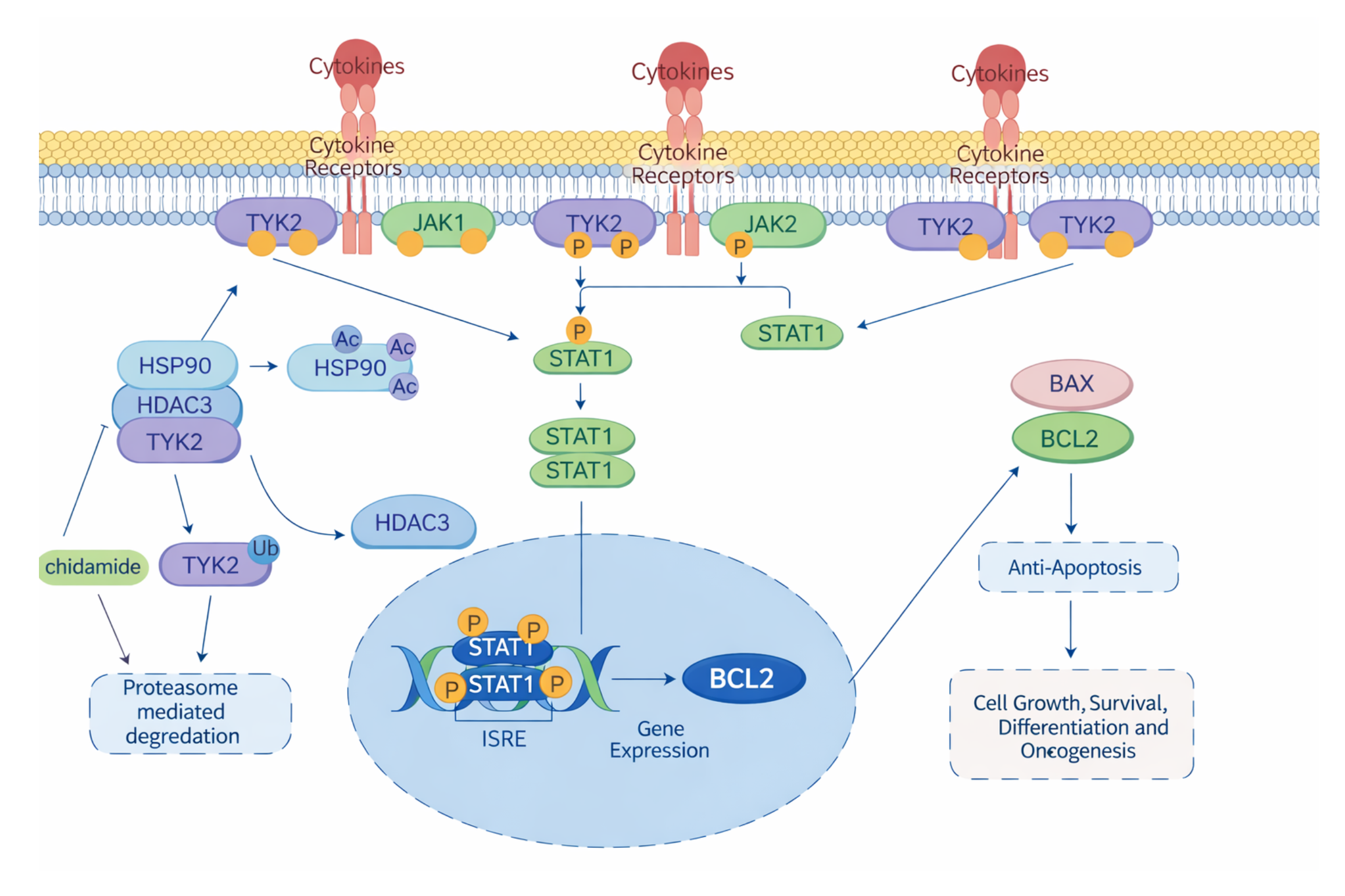


**Figure S8.** Schematic illustration of the proposed mechanism of chidamide regulating the HDAC3-HSP90-TYK2-STAT1-BCL2 signaling model in T-ALL. Cytokine stimulation activates cytokine receptors and the JAK-TYK2 complex, leading to phosphorylation of STAT1. Phosphorylated STAT1 dimerizes and translocates into the nucleus, where it binds interferon-stimulated response elements (ISREs) and contributes to the transcription of survival-associated genes, including BCL2. Based on the results of the present study, HDAC3 is involved in the regulation of TYK2 stability, potentially through interaction with the molecular chaperone HSP90. Inhibition of HDAC3 by chidamide is associated with increased HSP90 acetylation, enhanced ubiquitination of TYK2, and subsequent proteasome-mediated degradation of TYK2, leading to attenuation of STAT1 activation. Consistent with previous reports demonstrating that HSP90 inhibition induces TYK2 degradation and apoptotic cell death in T-ALL (Akahane K et al., Leukemia. 2016), reduced TYK2-STAT1 signaling is associated with decreased BCL2 expression and a shift toward pro-apoptotic signaling.
